# Mesoporous Silica Nanoparticle-Based Imaging Agents for Hepatocellular Carcinoma Detection

**DOI:** 10.3389/fbioe.2021.749381

**Published:** 2021-11-16

**Authors:** Xuqi Peng, Gan Lin, Yun Zeng, Zhao Lei, Gang Liu

**Affiliations:** State Key Laboratory of Molecular Vaccinology and Molecular Diagnostics, Center for Molecular Imaging and Translational Medicine, School of Public Health, Xiamen University, Xiamen, China

**Keywords:** mesoporous silica nanoparticles, hepatocellular carcinoma, diagnostics, multimodal imaging, biomedical applications

## Abstract

Hepatocellular carcinoma (HCC) is characterized by poor prognosis and high mortality. The treatment of HCC is closely related to the stage, and the early-stage of HCC patients usually accompanies a more long-term survival rate after clinical treatment. Hence, there are critical needs to develop effective imaging agents with superior diagnostic precision for HCC detection at an early stage. Recently, mesoporous silica nanoparticles (MSNs) based imaging agents have gained extensive attentions in HCC detection, which can serve as a multifunctional nanoplatform with controllable size and facile surface functionalization. This perspective summarizes recent advances in MSNs based imaging agents for HCC detection by the incorporation of several clinical imaging modalities. Multi-modal imaging system has been developed for higher spatial resolution and sensitivity. Even though some limitations and challenges need to be overcome, we envision the development of novel MSNs based imaging agents will offer great potential applications in clinical HCC detection.

## Introduction

Liver cancer incidence continued to increase, and the 5-years relative survival rate of liver cancer is only 20% (Siegel et al., 2021). Hepatocellular carcinoma (HCC) is the fourth most common cause of cancer death worldwide, which is characterized by poor prognosis and high mortality (Villanueva, 2019). The diagnosis of HCC in clinic mainly including clinical features, diagnostic imaging and liver biopsy (El-Serag et al., 2008). However, most HCC patients are diagnosed at late and advanced stages, leading to undesirable treatment outcomes (Befeler and Di Bisceglie, 2002; Shi et al., 2018; Zhang et al., 2017). Precise diagnosis of HCC at an early stage is beneficial for choosing better treatment options. Certain new imaging techniques have been applied for precise HCC detection and improving the prognosis of patients, including ultrasound (Lin X. et al., 2019), computed tomography (CT) (Chen et al., 2014; Cheng et al., 2014), magnetic resonance imaging (MRI) (Cai et al., 2020; Wang C. et al., 2019), fluorescence imaging (Lin H. et al., 2019; Sun et al., 2019), photoacoustic imaging (PAI) (Lei et al., 2020; Zhou et al., 2021) and positron emission tomography imaging (Hu et al., 2012). Each imaging technique showed unique advantages and limitations for liver cancer screening, detection or intraoperative navigation. (Ariff et al., 2009). US imaging has been recommended as a screening imaging modality to screen liver cancer because of its simplicity, flexibility, non-invasive, low cost and real-time properties. However, the sensitivity of ultrasonic diagnosis for small liver cancer (<2 cm) was only 39–65%. The measurements of serum *α*-fetoprotein (AFP) is usually necessary to couple with Liver US for high-risk patients for early HCC screening. Once screening abnormal liver, dynamic enhanced CT and muti-modal MRI can be used for further diagnosis and staging due to their relatively higher sensitivity and specificity. Although MRI is particularly well-suited for liver cancer detection, it has not yet been used for intraoperative surgical guidance owing to its high cost and long imaging times. Fluorescence image has long been used for guiding surgery in liver cancer theranostics (Chen H. et al., 2020). For example, intraoperative NIR-II imaging with suitable fluorescence probes showed a high tumor-to-normal-liver-tissue signal ratio (5.33) during the fluorescence-guided surgical resection of liver tumors (Hu et al., 2020). Compared with fluorescence, PAI, an emerging new imaging method, which offers deeper penetration, can improve imaging contrast and spatial resolution of superficial tissue. Thus PAI is expected to be a new method for early diagnosis and staging of early liver cancer in the near future (Fu et al., 2019).

Nanoparticles (NPs) could integrate diagnostic and therapeutic agents into one nanosystem in the field of cancer diagnostics and therapy. For instance, lipid micelles could act as drug carriers, and many lipid-based NPs have been approved by Food and Drug Administration (FDA) for clinical tumor treatments (Mitchell et al., 2021). Apart from lipid, mesoporous silica nanoparticles (MSNs), as a class of inorganic materials, has been widely researched as drug/gene/molecular carriers for cancer theranostic (Lee et al., 2011; Li et al., 2019; Wu et al., 2011). Compared with lipid micelles, MSNs are precisely formulated with controllable particle size, morphology and structure. As liver is the main organ that sequesters majority of nanoparticles from blood circulation, nanoparticles can intrinsically target liver. From this point, MSNs can enhance the liver accumulation of imaging agents and enable effective liver targeting. Furthermore, MSNs modified with targeting ligand on the surface could accumulate in the tumor tissues by active targeting (Chen F. et al., 2013; Goel et al., 2014; Li et al., 2018a; Rosenholm et al., 2009; Zhang J. et al., 2013). Since the size of nanomaterials below 200 nm could accumulate in solid tumors via the enhanced permeability and retention (EPR) effect (Matsumura and Maeda, 1986). The MSNs with size between 50 and 200 nm could accumulate in tumors as the leaky vessels enable NP extravasation, which is also called passive targeting strategy (Chen Z. et al., 2020; Li et al., 2016; Meng et al., 2011). Graft PEG or its derivatives onto the surface of MSNs can not only stabilize the NPs in the aqueous solution, but can escape opsonization in the blood. The 50-nm MSNs coated with PEI-PEG copolymer showed passive accumulation of about 12% at the tumor site, only 3% when coating with a 5 kD PEG polymer (Tang et al., 2012). Besides, the excellent biocompatibility and low hemolytic activity of MSNs offers promising potential in clinical applications (Asefa and Tao, 2012; Chen Y. et al., 2013; Tarn et al., 2013). These advantages offer enormous opportunities for designing MSNs-based nanocomposites for bioimaging and cancer therapy (Chen et al., 2016; Fan et al., 2014; Hong et al., 2017; Liu S. et al., 2020; Singh et al., 2012). Inspiringly, the multifunctional theranostic system can control the quantity of drug molecular and imaging contrast agents simultaneously for effective and safe theranostics. (Zhao et al., 2012). Multimodal imaging, compared with single imaging modal, showed higher spatial resolution and sensitivity. And multimodal imaging-guided treatments have been developed for precision synergistic cancer therapy (Sun et al., 2018). Herein, we focus on the recent advances of MSNs based imaging agents for HCC detection. The intent is to give the readers a critical discussion of the design and applications of MSNs based imaging agents.

## Mesoporous Silica Nanoparticles

With the dramatic development of nanotechnology and nanomedicine during past decades, oceans of organic/inorganic nanomaterials have been designed for cancer therapy and diagnosis. Especially, benefit from the unique qualities of tunable particle size, large pore volume, high surface area, facile surface functionalization, as well as excellent biocompatibility and biodegradation, MSNs has been produced as nanocarriers for the art of cancer diagnostics (Caltagirone et al., 2015; Montalti et al., 2014; Ni et al., 2017; Tang et al., 2012). For biomedical applications, the size and morphology of MSNs are critical for cellular uptake (Zhang et al., 2009). It was estimated that the maximum uptake size of MSNs by cells was the diameter of 50 nm (Lu et al., 2009). The rates of endocytosis for MSNs with different morphology were similar for CHO cells, while differed for fibroblast cells (Trewyn et al., 2008). Besides, the cytotoxicity (Yu et al., 2011), biodistribution (Fu et al., 2013; He et al., 2011; Zhao et al., 2016) and excretion (Croissant et al., 2017) of MSNs are also attracted much attention. The silica particle size of 100–500 nm showed low cytotoxicity (Hadipour Moghaddam et al., 2019), and the number of silanol groups affects the hemolytic properties (Slowing et al., 2009). MSNs were determined mainly distributed in the liver, spleen and lung after intravenous injection into mice (Huang et al., 2011). MSNs with negative charge showed higher uptake and retention in the liver, while positive charged MSNs undergo rapid hepatobiliary excretion and transport into the gastrointestinal tract (Souris et al., 2010). In this section, we summarized the synthesis method of MSNs and the way of controlling the pore size/volume of MSNs.

### Design and Synthesis of MSNs

With over 20-years development since discovered by Kuroda’s and Kresge’s groups in 1990s (Yanagisawa et al., 1990), MSNs possess certain unique advantages, such as extensive and uniform mesoporosity, tunable particle size (10–1,000 nm), wide-range pore diameter (2–20 nm), flexible morphology and so on. Mesoporous silica is usually produced by a surfactant-directing method under basic, acidic or neutral conditions. Silica precursors are first hydrolyzed and combined with the head groups of surfactants by either electrostatic force or hydrogen bond interaction to form a liquid-crystalline mesophase. Then the transformation of mesophases occurs during the hydrolysis and condensation of silica species. The interactions between surfactants and silica precursors, as well as the rate of hydrolysis and condensation of silica species which heavily depend on the pH value of the reaction system, directly affect the formation and the morphology of mesophases (Narayan et al., 2018).

Silica nanoparticles with good monodispersity were synthesized by catalyzing tetraethyl orthosilicate with ammonia by stöber in 1968 (Stöber et al., 1968). Since then, many researchers reported the MSNs with various sizes from nano to micro via modified stöber method (Hao et al., 2015; Slowing et al., 2007; Taylor et al., 2008; Wan and Zhao, 2007; Wu et al., 2013). And several groups committed to design and synthesis superb MSNs. For example, Zhao’s group prepared well-ordered hexagonal mesoporous silica structures (SBA-15) using triblock copolymer (Zhao et al., 1998). Bein and co-workers presented a general method for the preparation of highly dispersed MSNs ranging in size from 40 to 150 nm (Kobler et al., 2008). Subsequently, Shi’s group reported the MSNs with the uniform size of 25–105 nm by following Bein’s protocol with a certain modification (Pan et al., 2012). Nanopore-engineering strategy endows functional materials with versatile moieties and promotes the development of various imaging modalities.

### Controlling of Pore Size/Volume

Biofunctional materials encapsulated into the cavities or the mesopores of MSNs to fabricate potential nanotheranostic platforms. The pore sizes/volumes of MSNs influence the effect of therapy or diagnostics. For example, MSNs with large pore volume possessed high loading capacities for anti-cancer drugs, and showed enhanced cytotoxicity (Li and Shi, 2014). As a matter of fact, the pore sizes of these mesoporous nanostructures are usually very small (2–5 nm), resulting from the use of cetyltrimethyl ammonium bromide (CTAB) or other alkylammonium surfactants as templates, which greatly hindered their further bio-applications in the encapsulation of biomacromolecules (Slowing et al., 2006; Xuan et al., 2016). Therefore, many new approaches have been developed to enhance the drug storage capacity of MSNs (Feng et al., 2016; Trewyn et al., 2007; Vallet-Regi et al., 2007; Vivero-Escoto et al., 2010).

One way to adjust the pore size is that, utilizing of a swelling agent during the synthetic process of MSNs, the pore sizes of MSNs were expanded up to 45 nm for higher protein loading capacity by mixing oil phase (chlorobenzene and tetraethyl orthosilicate) (Xu et al., 2015). The pore structure of MSNs was formed through a supramolecular self-assembly mechanism, which could be influenced by the oil/water ratio, stirring rate, and growth time in the synthetic process. Shi et al. synthesized monodispersed, large-pore silica nanospheres, with three different mesostructures (hexagonal, cubic and lamellar) by adjusting the amount of CTAB (Niu et al., 2014). With the increase of the concentration of CTAB, the micelles morphologically transformed from lamellar to rod-like or spherical. Subsequently, the formed micelles were sell-assembled to the ordered long period stacking mesostructures. A study reported the monodispersed MSNs with large pores, using 1,3,5-Trimethylbenzene as pore swelling agents (Kim et al., 2011). The mean pore sizes of these MSNs were increased dramatically from 2.1 to 23 nm. The pore structure of stellate, raspberry, or worm-like morphologies was developed based on the nature and the concentration of small organic amines together with an appropriate choice of the cationic surfactant counterions (Zhang K. et al., 2013).

Besides, hollow MSNs were designed for increasing the pore volume to improve the loading capacity (Hu et al., 2011), and had been used in many research fields, such as catalysis (Fang et al., 2013; Mahmoud et al., 2013), drug/gene delivery (Li et al., 2017; Ma et al., 2013; Slowing et al., 2008) and bioimaging (Lou et al., 2008; Lv et al., 2014; Zhao and Jiang, 2009). For example, Yang et al. reported a new type of hollow MSNs (HMSNs) with enhanced loading capacity, and fabricated the nanocomposite of FePt nanoparticles for evaluating the therapy and diagnostics effects on Hela cells (Lin et al., 2017). The nanocomposite showed synergistic anticancer effects and could serve as dark T2 contrast agent for MRI. In another research, the rattle-type mesoporous silica hollow spheres were reported by using a selective etching strategy (Chen et al., 2009). The silica nanorattles were synthesized by etching the middle layer of the silica framework.

## Mesoporous Silica Nanoparticle Based Diagnosis

### Ultrasound Imaging

Ultrasound (US) imaging is the most common and primary technique to screen many different diseases, because of its simplicity, flexibility, non-invasive and low cost (Kiessling et al., 2014). US contrast agents are usually comprised of several micro gas bubbles, which is stabilized by shell made of lipids, proteins or polymers, which can improve the definition and resolution of ultrasonic image (Sirsi and Borden, 2009). The main shortcomings of microbubbles are their micron size and poor stability, which hinder their applications for diagnosis. To cope with this, MSNs-based US contrast agents has been developed and generated bubbles with lifetime of 30 min at least (Jin et al., 2017). By actively targeting strategies, MSNs could serve as promising US contrast agents for HCC diagnosis. Silica-based US imaging agents can conjugate with epithelial cell-adhesion molecule (EpCAM) aptamer as targeted diagnostics agents for the HCC cell line (HepG2) (Pilapong et al., 2017). The US signal showed an enhancement effect *in vitro* due to the extraordinary mesoporous structure. Meanwhile, considering the high levels express of glypican-3 protein (GPC-3) on HepG2 cells, it has been deemed as a marker of HCC. For instance, the GPC-3 ligand peptide-functionalized silica nanoparticles demonstrated significantly enhanced ultrasound intensity for HepG2 cells ultrasound molecular imaging (Di Paola et al., 2017).

With the characteristics of real-time and inexpensive, US has been reported in guiding therapy as well (Wang et al., 2013). After intravenous injection of US imaging agent in HCC tumor-bearing nude mice, the US imaging showed brightened in tumor tissues. The nanoparticles accumulated in tumor tissues, and this phenomenon can be explained by the EPR effects. In general, silica-based US imaging agents reduced the toxicity of gas release in traditional gas-filled microbubbles and it is facile to functionalize targeted agents or drugs on the surface of MSNs. However, the current research mainly focused on the enhancement of the ultrasound contrast for cells, the distribution, metabolism, specificity and sensitivity of agents in the animal model need more accurately evaluation.

### Photoacoustic Imaging

PAI integrates optical and ultrasound imaging, a non-ionizing imaging modality, and offers deeper penetration than pure optical imaging and richer optical contrasts than US imaging (Kim et al., 2016). Hence, PAI has attracted much attention in biological tissues detection. Hyaluronate derivatives have been investigated as a target-specific delivery to liver, owing to the overexpression of cluster determinant 44, which was regarded as the HA receptor. Lee et al. developed a hyaluronate–silica nanoparticle containing a certain amount of nitrogen atoms as a liver targeting PA contrast agent, which showed high liver-specific targeting efficiency (Lee et al., 2018). Nitrogen atoms play to the formation of nonbonding or defective sites, the sites between the bandgap of silica nanoparticles result in the increase of light absorption. The boundary of the liver in the PA imaging showed highly clear after intravenous injection of contrast agent for 12 h. This novel PA contrast agent could provide more details. Li and co-workers reported a novel 2-dimension composite nanoplatform by coating a thin mesoporous-silica shell onto the surface of Ti_3_C_2_ MXene for PA imaging, the composite nanosheets showed excellent optical and ultrasound imaging effects grounded on the localized surface plasmon resonance effect (Li et al., 2018b). Furthermore, doxorubicin was loaded into the nanostructures for synergistic chemotherapy and photothermal therapy. The *in vivo* PA imaging demonstrated that the composite nanosheets could be applied as PA contrast agents for real-time monitoring of the therapeutic process. More recently, Chaudhary et al. explored a series of indocyanine green (ICG) loaded MSN-based nano-carriers with various surface modifications for PAI (Chaudhary et al., 2019). The ICG loaded layer-by-layer polyelectrolyte coating MSN (LBLMSN-ICG) exhibited 4-times enhanced PA signal *in vitro* in comparison with the same concentration of pure ICG.

The mesosilica materials not only acted as carriers, but endows the 2D sheets with well-defined mesopores for multimodal diagnostic and therapy. The as-synthesized nanocomposites still need carefully control on the thickness of silica-shell and the uniformity of mesopore structure.

### Fluorescence Imaging

As a classic near infrared (NIR) fluorescence imaging agent, ICG has been approved by FDA for clinical applications in 1958 (Frangioni, 2003). ICG is a low toxic and injectable NIR organic dye and has been widely used in clinical imaging, especially for precise surgical resection. (Quan et al., 2012). However, there are still some drawbacks that limit its biological applications, such as low fluorescence quantum yield in aqueous solutions, poor solubility in physiological aqueous conditions (Desmettre et al., 2000; Saxena et al., 2003).

Benefited from the high chemical stability and superb drug loading ability of MSNs, Tian and others developed arginine-glycine-aspartic acid (RGD)-conjugated MSNs loaded with ICG for imaging-guided surgery (Zeng et al., 2016). And the RGD-conjugated MSNs could identify precise tumor margin (1 mm) during liver cancer surgery. Furthermore, the microtumor lesions (0.4 ± 0.21 mm) showed excellent optical contrast under NIR and GFP fluorescence images. ICG has been used as a near-infrared photothermal therapy reagent for its high photothermal conversion efficiency. More recently, Chang’s group reported a multifunctional (ICG + sorafenib)@mSiO_2_ nanosystem for highly efficient synergistic diagnosis and treatment of HCC (Yang et al., 2020). The nanosystem showed outstanding real-time fluorescence imaging, which was responsive to 808 nm laser irradiation. Notably, the silica nanosystem endows the ICG with a higher level of endocytosis and longer red fluorescence signal retention.

Besides, fluorescent conjugated polymers is another choice for fluorescence imaging agent, because of their high quantum efficiency, good photostability, and fast radiative rate (Feng et al., 2008). An efficient controlled release system has been realized by a biofunctional nanocomposite for pH-controlled drug delivery and cellular imaging simultaneously, capping with polyelectrolytes at the outer surface of MSNs (Pu et al., 2013). Hydrophobic conjugated polymers have been converted into dispersible in the aqueous environment by silica encapsulation strategies, which makes them excellent candidates for fluorescence probes of HCC cells (Tan et al., 2011). The conjugated polymers with different emissions can be realized by employing other fluorescent conjugated polymers to emit a blue, green, yellow, and red color. Fluorescent conjugated polymers could be a powerful tool for cell imaging with silica encapsulation strategies.

Moreover, MSNs were decorated with Aggregation Induced Emission (AIE) fluorogen PhENH_2_ and MoS_2_ nanosheets for both tumor diagnosis and treatment (Wang J. et al., 2019). The AIE fluorogens which chemically modified on the surface of MSNs showed more stable fluorescence than physically absorbed luminescent molecules. As for fluorescent bioprobes, the challenge is how to solve the problem of frequently quenching in high concentrations or aggregate states. AIE-based materials shed new light on fluorescence imaging.

### Magnetic Resonance Imaging

MRI is recognized as the most popular technology for evaluating the liver tissue owing to its ultrahigh sensitivity and specificity (Bellin et al., 2003). Gadolinium chelates, as a non-specific extracellular contrast, are used extensively for liver MRI in clinical medicine.

In order to enhance MRI imaging effect, both passive and active targeting strategies have been adopted to increase the accumulation of imaging agents in tumors. In consideration of the very short blood circulation time of Gd-EOB-DTPA, loading Gd (III) chelates into MSNs could act as a promising T_1_ MRI imaging contrast agent for cancer diagnostics (Vivero-Escoto et al., 2011). Although there are still great concerns about the possibility of nephrogenic systemic fibrosis resulting from the Gd^3+^ ions, this platform provides a choice for alleviating the safety concerns (Broome, 2008; Rydahl et al., 2008). Recently, Mn-doping MSNs has been reported as the contrast agent for T_1_-weighted MRI, with the accumulation in the tumor confirmed due to the EPR effects (Yu et al., 2016). The manganese-doped hollow MSNs were sensitive to tumor microenvironment, resulting in the breaking up of the Mn-O bond and releasing of manganese ions. To a certain extent, the “manganese extraction” strategy ravel out concerns about the biodegradation of inorganic mesoporous nanosystems and provides a fresh idea for biodegradation of inorganic material nanofamily. In order to address the limitations of non-specific contrast agents, Kim et al. investigated the liver-specific MRI contrast agent, Mn^2+^-doped SiO_2_ nanoparticles (Mn-SiO_2_), enhancing the visibility of HCC lesion (Kim et al., 2013). The liver-to-HCC MR contrast ratio could be used for the conspicuous detection of HCC. The maximum signal enhancement of the liver parenchyma was observed in the images after intravenously administered via the tail vein for 6 h. They proposed that the nanoparticles engulfed in Kupffer cells would release the Mn^2+^ ions, thus T_1_-weighted MRI showed hyperintense in healthy liver tissues with abundant Kupffer cells over lesions.

Superparamagnetic iron oxide (SPIO) particles, acting as a negative contrast agent, were used in liver-specific contrast imaging (Lee et al., 2007; Na et al., 2009). Uniform magnetic MSNs were prepared by coating Fe_3_O_4_ core with mesoporous silica shells, which could be used for the enhancement of T_2_-weighted MR imaging (An et al., 2015). Benefit from the easily surface functional of silica coating layer, magnetic MSNs can be modifed with folic acid for targeted delivery and real-time tumor monitoring. (Chi et al., 2019).

Otherthan the passive and active targeted by modified with cellular receptors, magnetic targeting technology is a very attractive physical targeting technology (Hajba and Guttman, 2016; Tian et al., 2014). Magnetic MSNs could be magnetically targeted to tumor tissues in HepG2 xenograft-bearing nude mice using small NdFeB permanent magnets (Wang et al., 2018). Similarly, magnetic field not only accumulated MSN-coated iron oxide nanoparticles, but also controlled the “OFF-ON” state of the magnetic drug delivery system (Liu J. et al., 2020). SPIOs act as an excellent biocompatible inorganic material, on which various thicknesses of silica coating can be realized for enhanced MR imaging. But the concern is that, SPIOs are no longer clinically used for MRI agents as a result of some drawbacks. More efforts on magnetic MSNs for clinical transformation could be in vain until SPIOs were re-approved by FDA.

### Computed Tomography

CT is one of the most common modalities for diagnostics of disease or cancer since its rapid image generation and low cost (Foley et al., 2000). Small iodinated molecules, usually used as CT contrast agents in clinical, experienced fast renal excretion and short imaging time hinder their applications (Kim et al., 2010). Gold nanoparticles have attracted prominent interests owing to their strong X-ray attenuation properties, and have been widely used as CT contrast agents (Pelaz et al., 2017). Dong’s group developed a Janus-structured gold-MSNs act as targeted CT-imaging agents for HCC diagnosis (Wang et al., 2017). *In vivo*, the Janus nanoparticles exhibited a clearly distinguished CT signal in the corresponding tumor site of the tumor-bearing nude mice. The unsymmetrical structures of Janus nanoparticles provide higher radiation-absorption efficiency. The tumor targeted MSNs with gold gatekeeper showed higher CT signal intensity because of its EPR effects as well as active targeting through EpCAM receptor (Babaei et al., 2017). Qin et al. reported dual-MSNs containing small mesopores and large mesopores for gold nanorices encapsulation (Qin et al., 2019). They confirmed that multiple gold nanorices (GNRs) in a nanoscale matrix showed brighter CT signals with the increase of Au concentrations, and the hounsfeld unit values of MSN-based GNRs were higher than individual GNRs. These as-prepared nanocompound possess the potential for application in CT imaging and imaging-guided photothermal therapy. Mesoporous silica could coat on Au NPs for improving its hydrophilicity and drug loading dose. However, there is nearly no report about the relationship between CT signals and the thickness of silica coating for HCC diagnostic.

### Multimodal Imaging

Multimodal imaging has been developed to satisfied the requirement of both high spatial resolution and high sensitivity for imaging diagnosis (Cutler et al., 2013; Lee et al., 2012; Sailor and Park, 2012; Zhang et al., 2015). Recently, multifunctional nanoparticles have been explored as multimodal imaging nanoprobes (Chu et al., 2020; Liu et al., 2018), or used for imaging-guided tumor therapy (Chen et al., 2018; Li et al., 2021; Lin et al., 2018; Shi et al., 2019; Song et al., 2015; Wang et al., 2015; Zhu et al., 2019), or monitored therapeutic response (Ye et al., 2018). For instance, nanocomposites integrating near-infrared fluorescence with MRI and PAI exhibit ultrasensitivity, precise anatomical localization, and good spatial resolution for tumor detection (Wang et al., 2015).

As for HCC imaging, fluorochrome and iron oxide were loaded into silica nanoparticles for MRI and optical dual-mode imaging (Liu et al., 2021). And this dual-mode imaging was developed for a preoperative diagnosis of tumor cells and improving the spatial resolution during surgery (Tan et al., 2011). Conjugated polymers showed excellent fluorescent properties and could use as fluorescent labelling agents for cellular imaging. The nanocapsules combined conjugated polymers as the fluorescent emitter and superparamagnetic iron oxide nanoparticles as the T_2_ enhanced MRI contrast agents. Both confocal microscopy images and MRI showed that the cellular uptake of the nanocapsules was enhanced by the external magnetic field. Over and above the fluorescent conjugated polymers, upconversion nanoparticles (UCNPs) as excellent UC luminescence agents have been used for accurate cancer diagnosis. The multifunctional composite combined with MnFe_2_O_4_ and UCNPs were magnetic guidance to the tumor for *in vivo* MR and UC luminescence imaging (Ding et al., 2019). Magnetic gadolinium oxide-iron oxide with mesoporous silica shell, presented the r1 and r2 values were 10 mM^−1^ s^−1^ and 165 mM^−1^ s^−1^, separately, and could be used as a T_1_ and T_2_ dual mode contrast agent. (Das et al., 2018). Inorganic Janus nanoparticle with extraordinary heterostructure could guide the cancer therapy by CT/MR imaging *in vivo* (Zhang et al., 2018).

Besides, triple-mode imaging agents including MRI, US, and fluorescence were developed for HCC cell lines imaging (Pilapong et al., 2017). The nanoprobe determined that the r2 value were 110.9 mM^−1^ s^−1^, and the porous structure of which enhanced US signals and reduced the side effects of gas release in conventional gas-filled microbubbles. The MRI/US/fluorescence imaging probe could visualize the microscopic-scale and macroscopic-scale of the HepG2. Another research focused on triple-mode upconversion luminescence (UCL)/CT/MR imaging-guided synergistic chemo-photothermal therapy of HCC (Chen X. et al., 2020). However, it was hard to maximize the diagnostics and treatment effects on “all in one” nanoplatforms, simultaneously.

**FIGURE 1:**
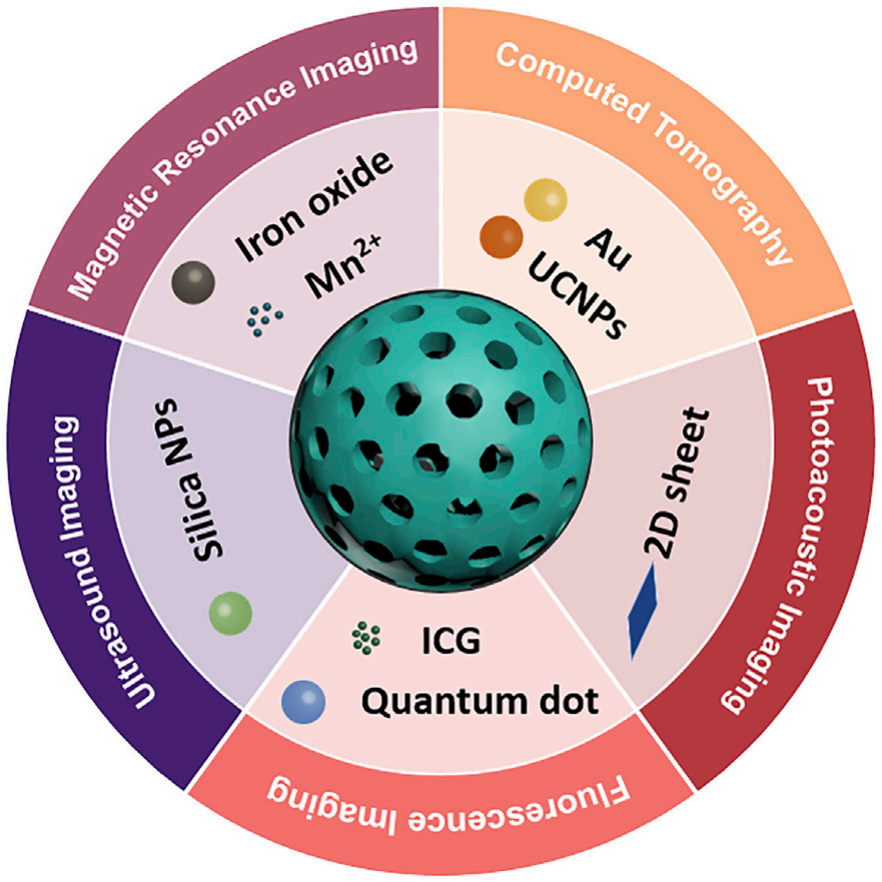
Different imaging modalities based on MSNs.

Although more and more muti-model imaging agents were developed for precise diagnosis of HCC, the effect of combined agents still need deeper exploration. And the choice of suitable imaging modalities is significant for the art of an “all in one” system. Multifunctional nanoplatform can be designed for multimodal imaging guided multiple therapies. For example, tetra-modal imaging agents containing CT, MRI, UCL and photothermal imaging were produced and applied as drug carriers for multiple anticancer therapies (photothermal, photodynamic therapy, and chemo-therapy), simultaneously (Lv et al., 2015). As showed in Figure 1, MSNs based imaging agents could use in different imaging modalities. Table.1 summarized the MSNs-based nanomaterials for HCC imaging.

**TABLE 1:** MSNs-based nanomaterials for HCC imaging.

Imaging model	Nanomaterials	Size (nm)	DLS (nm)	Polydis-persity index	Zeta potential (mV)	Surface area (m^2^/g)	Pore size (nm)	Pore volume (cm^3^/g)	Targeting moiety	Type of cells	Animal models	Concentration of NPs	References
US Imaging	mSiO_2_-MNP	9.4 ± 1.2	—	—	—	136.7	—	0.46	EpCAM aptamer	HepG2 cells	—	0.05 mg/well	Pilapong et al. (2017)
	GPC-3 SiNPs	250	450	—	−16 ± 1.7	—	—	—	GPC-3 targeting peptide	HepG2 cells	—	0.1 mg/ml	Di Paola et al. (2017)
	MSNC at Au-PFH-PEG	250	250	—	—	230	4.5	0.4	—	HCC cells	Rabbit VX2 xenograft; HCC tumor-bearing nude mice	6 mg/ml	Wang et al. (2013)
PA Imaging	HA–SiNP conjugates	1.99	—	—	—	—	—	—	Hyaluronate	HepG2 cells	Normal balb/c mouse	20 mg/ml	Lee et al. (2018)
	Ti_3_C_2_at mMSNs	—	116.4	—	−30	772	3.1	0.96	RGD	HCC cells	Tumor-bearing nude mice xenografts	5 mg/kg	Li et al. (2018b)
	LBLMSN-ICG	∼71	141.8 ± 51.3	0.64 ± 0.04	−17.98 ± 1.24	—	—	—	—	HepG2 cells	Male mouse cadavers	0.2 mg/ml	Chaudhary et al. (2019)
Fluo-rescence Imaging	ICG/MSNs-RGD	∼100	∼100	—	—	—	—	—	RGD	Hep-G2-GFP-fLuc	(Hep-G2-GFP-fLuc) xenograſts	0.2 mg/ml	Zeng et al. (2016)
	(ICG + S) at mSiO_2_	∼100	∼100	—	∼-17	—	—	—	—	H22 cells	Female C57BL/6J mice	1 mg/ml	Yang et al. (2020)
	Fluorescent Conjugated Polyelectrolyte-Capped MSN	∼100	204	0.22	—	920	2.5	—	—	HepG2 cells	—	0.28 mg/ml	Pu et al. (2013)
	C6PF BP-PPV PDHFBT MEH-PPV	26.7; 24.3; 25.1; 22.9	65.4; 51.4; 62.3; 51.3	0.169; 0.143; 0.18; 0.168	—	—	—	—	—	HepG2 cells	—	0.06 mg/ml	Tan et al. (2011)
	PhENH2-MoS2-FA MSNs	180	220	—	-35	—	—	—	Folic acid	HepG2 cells	—	0.0065 mg/ml	Wang et al. (2019b)
MR Imaging	PEGylation of Mn-HMSNs	100	141	—	—	222	3.8	0.53	—	HepG2 cells	Female BALB/c nude mice	2.5 mg/kg	Yu et al. (2016)
	Mn-SiO_2_	25	—	—	—	—	—	—	—	HepG2 cells	Orthotopic xenograft model	3 mg/kg	Kim et al. (2013)
	M-DMSN at pLAMA	250	—	—	—	119	—	0.08	Glycopolymer	HepG2 cells	Male BALB/c nude mice	100 mg/kg	An et al. (2015)
	M-LPMSNs	120	—	—	—	—	7	—	Folic acid	SMMC-7721 cells	Female BALB/c mice	2 mg Fe/cm^3^ tumor	Chi et al. (2019)
	S-M-MSNs	250	—	—	—	453.6	2.8	0.32	—	HepG2 cells	HepG2 xenograft-bearing nude mouse	25 mg/kg	Wang et al. (2018)
	R-M-MSNs	100*300	—	—	—	655.7	—	0.43	—	—	—	—	—
	IONP at MSN-N3	170	∼170	—	−30	767.17	3.04	0.576	—	HepG2 cells; HL-7702 cells	HepG-2 tumor-bearing mice	5.7 mg/kg	Liu et al. (2020a)
CT Imaging	GSJNs	225*110	—	—	—	758.5	2.4	0.51	Folic acid	SMMC-7721 cells; HL-7702 cells	SMMC-7721 xenografts nude mice	5 mg/ml	Wang et al. (2017)
	Au at Si-5-FU	—	48.27	0.2	11.43	191.6	2.9	0.35	EpCAM	HepG2 cells	HepG2 tumor-bearing nude mice	[Au] = 2 mM	Babaei et al. (2017)
	DMSSs	—	—	—	—	790.2	21.7	1.35	—	SMMC-7721 cells	SMMC-7721 xenografts nude mice	[Au] = 5.3 mg/ml	Qin et al. (2019)
Multi-modal imaging	Fe_3_O_4_ at PMO	362 ± 21	406		−31.4	539	2.9	0.694	—	HepG2 cells	Rabbit VX2 xenograft	0.02 mg/ml	Liu et al. (2021)
	UCMSs	∼50	—	—	−7	814	30	2.053	—	HepG2 cells	H22 tumor- bearing BALB/c mice	[Mn] = 0.6 mM	Ding et al. (2019)
	GdIO at mSiO_2_	40 ± 2	—	—	24	410.95	3.6	—	—	HepG2 cells	—	0.03 mg/ml	Das et al. (2018)

## Discussion

In this mini-review, we discuss the recent advances in MSNs based imaging agents for HCC detection. We summarize the design and synthesis of MSNs with different sizes and morphologies. Several imaging agents can be incorporated into MSN for HCC detection, including US, PAI, Fluorescence imaging, MRI, and CT. These agents have shown excellent imaging performance in HCC detection and encourage further exploration. Besides, MSN based multi-model imaging probes has also been developed for preoperative diagnosis of tumor tissues, enriching the information of imaging diagnosis. Despite great potentials of MSN-based imaging agents for HCC detection, there is numerous hurdles lying in the way of MSN-based imaging agents heading to the clinical translation. For example, large-scale production, quality control and standard characterizations are also needed for the commercialization of MSN-based imaging agents. The targeting of MSN-based imaging agents still needs improvement, as a recent analysis shows that only 0.7% of nanoparticles in the systemic route can reach tumor sites. The toxicity, particularly long-term toxicity aroused from nanoparticles, requires systematic and comprehensive *in vivo* studies. It is hopeful that with the constant efforts of scientists, radiologists and businessmen, MSN-based imaging agents can make a great breakthrough for disease diagnosis and benefit patients in the near future.

We hope that this mini review will provide readers with a better understanding on the design and synthesis of the MSNs for cancer imaging applications. More importantly, the design of MSNs based imaging agents for HCC detection should consider the imaging model, biocompatibility, and pharmacokinetics. Additionally, it is important to meet the clinical needs of HCC detection, developing imaging contrast agents with potential clinical translational capacity make it sense.

## Data Availability

The original contributions presented in the study are included in the article/supplementary material, further inquiries can be directed to the corresponding authors.
